# Supplementary material for: Increases in income-support payments reduce the demand for charity: A difference-in-difference analysis of charitable-assistance data from Australia over the COVID-19 pandemic
Source: PLoS One. 2023 Jul 12;18(7):e0287533. doi: 10.1371/journal.pone.0287533 (PMC10337872; doi:10.1371/journal.pone.0287533)
Supplement: S1 File — (DOCX) [file pone.0287533.s001.docx]

**Supplementary Information**

**Supplementary figures**

**S1 Fig.** Social expenditure as a percentage of GDP in OECD countries (2017)

**S2 Fig.** Unemployment benefits as a share of previous income (after 2 months, in %s)

**S3 Fig.** Timeline of key dates (2020-2021)

**S4 Fig.** Percentage change in assistance by charity (2019-2020)

**S5 Fig.** Percentage change in assistance by charity (2020-2021)

**S6 Fig.** Non-parametric regression discontinuity plot of the first AUD$750 Economic Support Payment

**S7 Fig**. Trends in assistance records (2020 vs. previous years)

**S8 Fig.** Coefficient plot from event study regression of time to and from nationwide shutdown in March 2020

**Supplementary tables**

**S1 Table.** Eligibility criteria for the Economic Support Payments and Coronavirus Supplement

**S2 Table.** Unstandardized coefficients from difference-in-difference regressions of daily assistance records (Full results)

**S3 Table.** Unstandardized coefficients from Poisson difference-in-difference regressions of daily assistance records (Full results)

**S4 Table.** Unstandardized coefficients from panel fixed effects regressions of daily assistance records

**S5 Table.** Results from a regression discontinuity analysis of the first Economic Support Payment

**S6 Table.** Unstandardized coefficients from triple-difference regressions of daily assistance records using The Salvation Army data (Full results)

**S7 Table.** Day and month corresponding to payment periods

**S1 Fig**. Social expenditure as a percentage of GDP in OECD countries (2017)


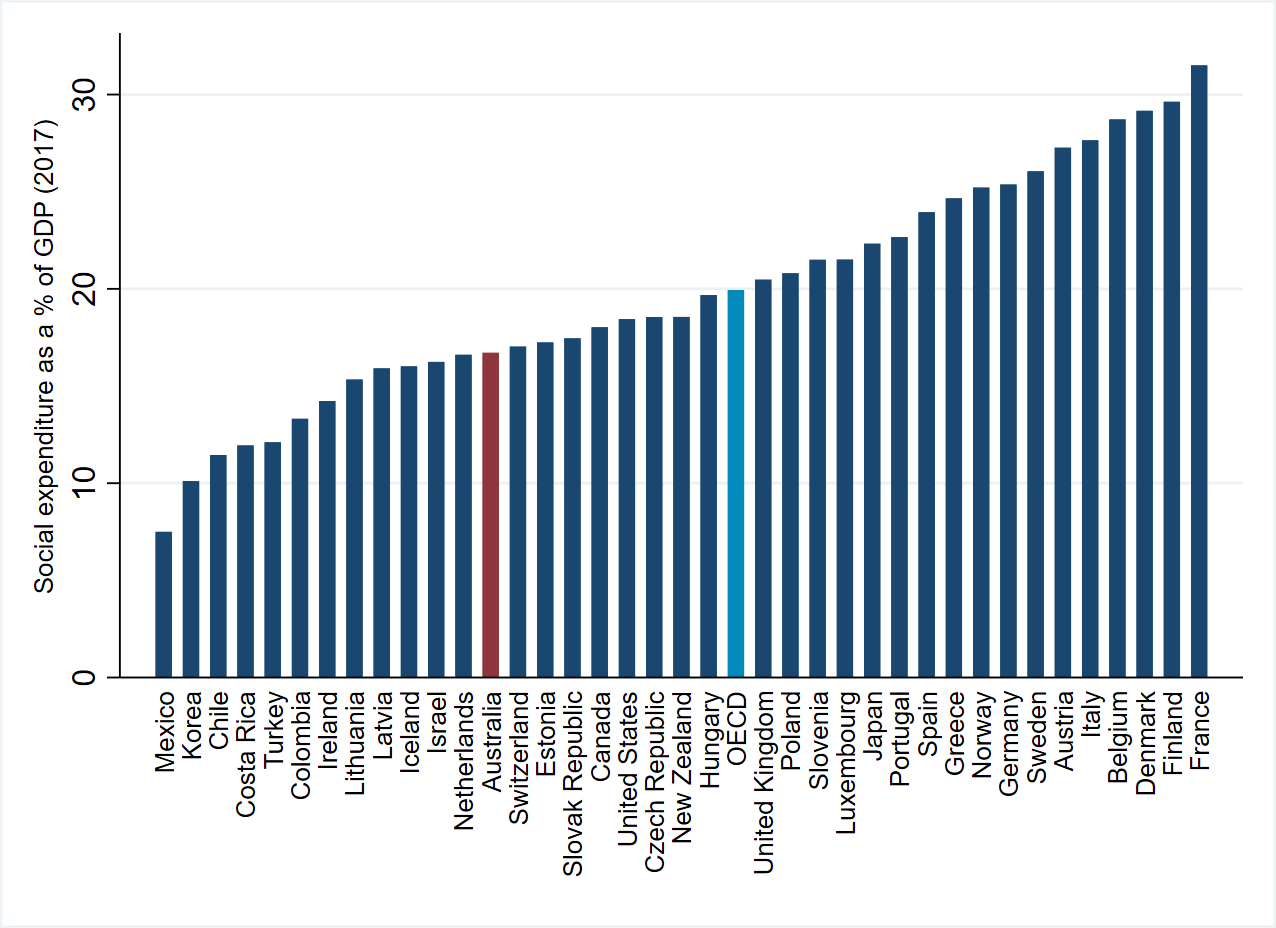


GDP – Gross Domestic Product; OECD – Organisation for Economic Cooperation and Development.

Source: OECD (2022), "Social Expenditure: Aggregated data", OECD Social and Welfare Statistics (database), https://doi.org/10.1787/data-00166-en (accessed on 1 April 2022).

**S2 Fig**. Unemployment benefits as a share of previous income (after 2 months, in %s)


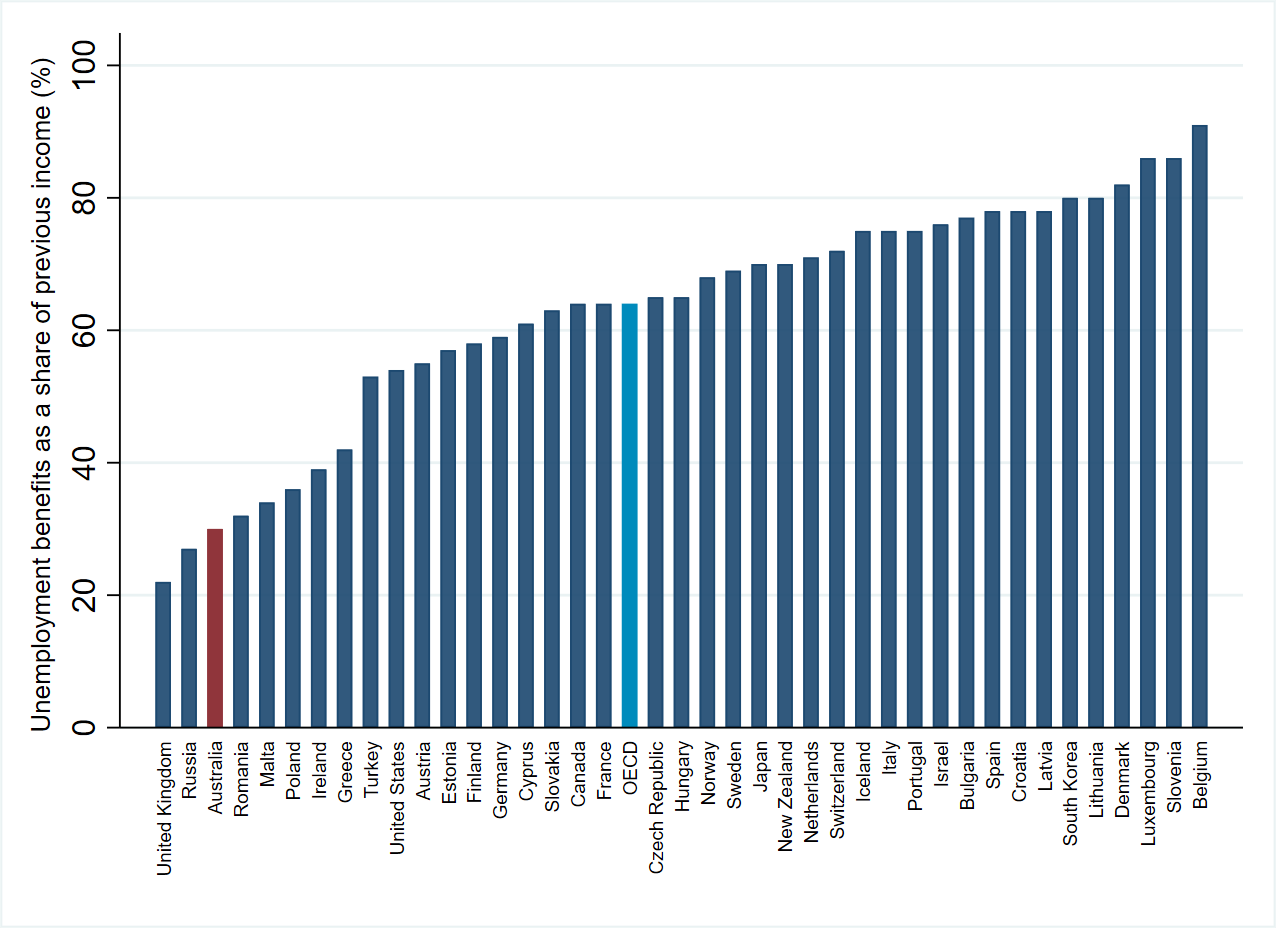


Figures are based on the latest available data per country.

Source: OECD (2022), Benefits in unemployment, share of previous income (indicator). doi: 10.1787/0cc0d0e5-en (Accessed on 1 April 2022).

**S3 Fig**. Timeline of key dates (2020-2021)


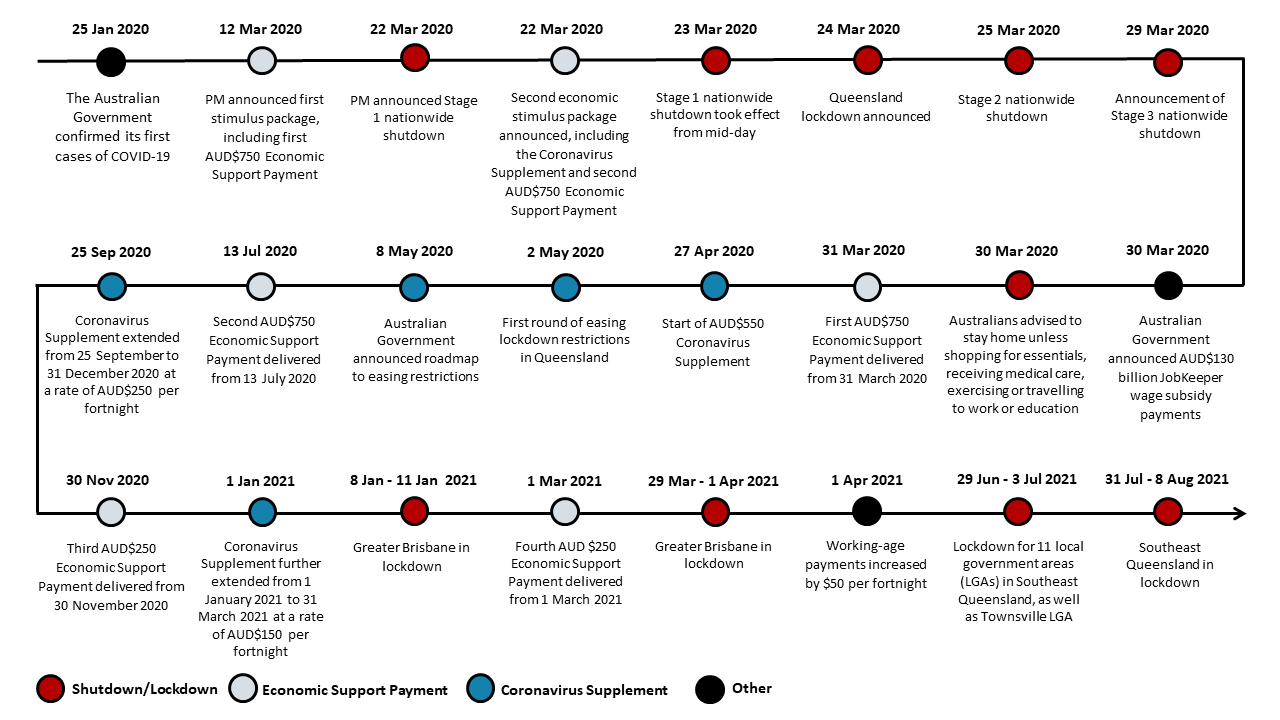


**S4 Fig**. Percentage change in assistance by charity (2019-2020)


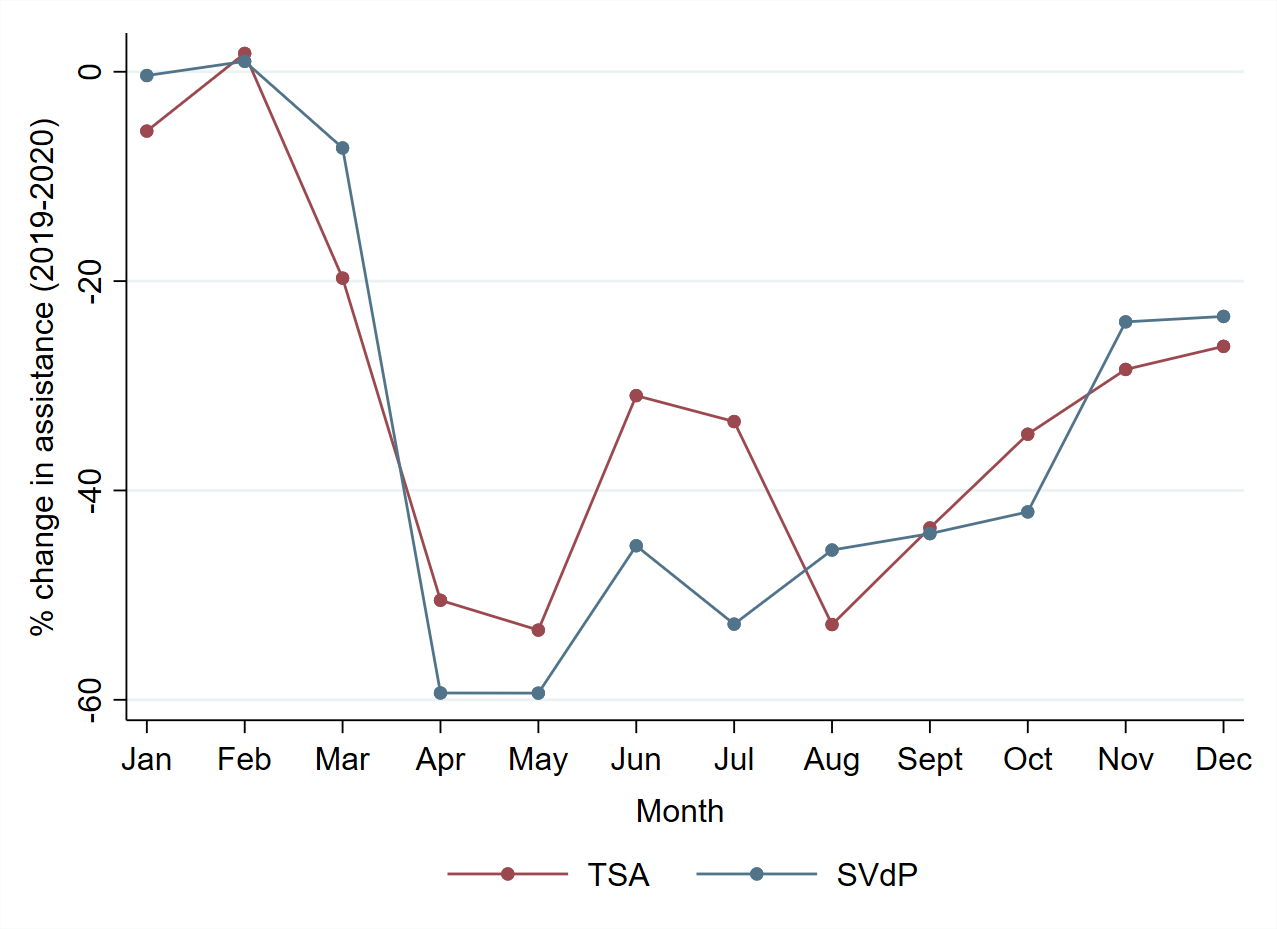


TSA – The Salvation Army; SVdP – St. Vincent de Paul Society Queensland.

**S5 Fig.** Percentage change in assistance by charity (2020-2021)


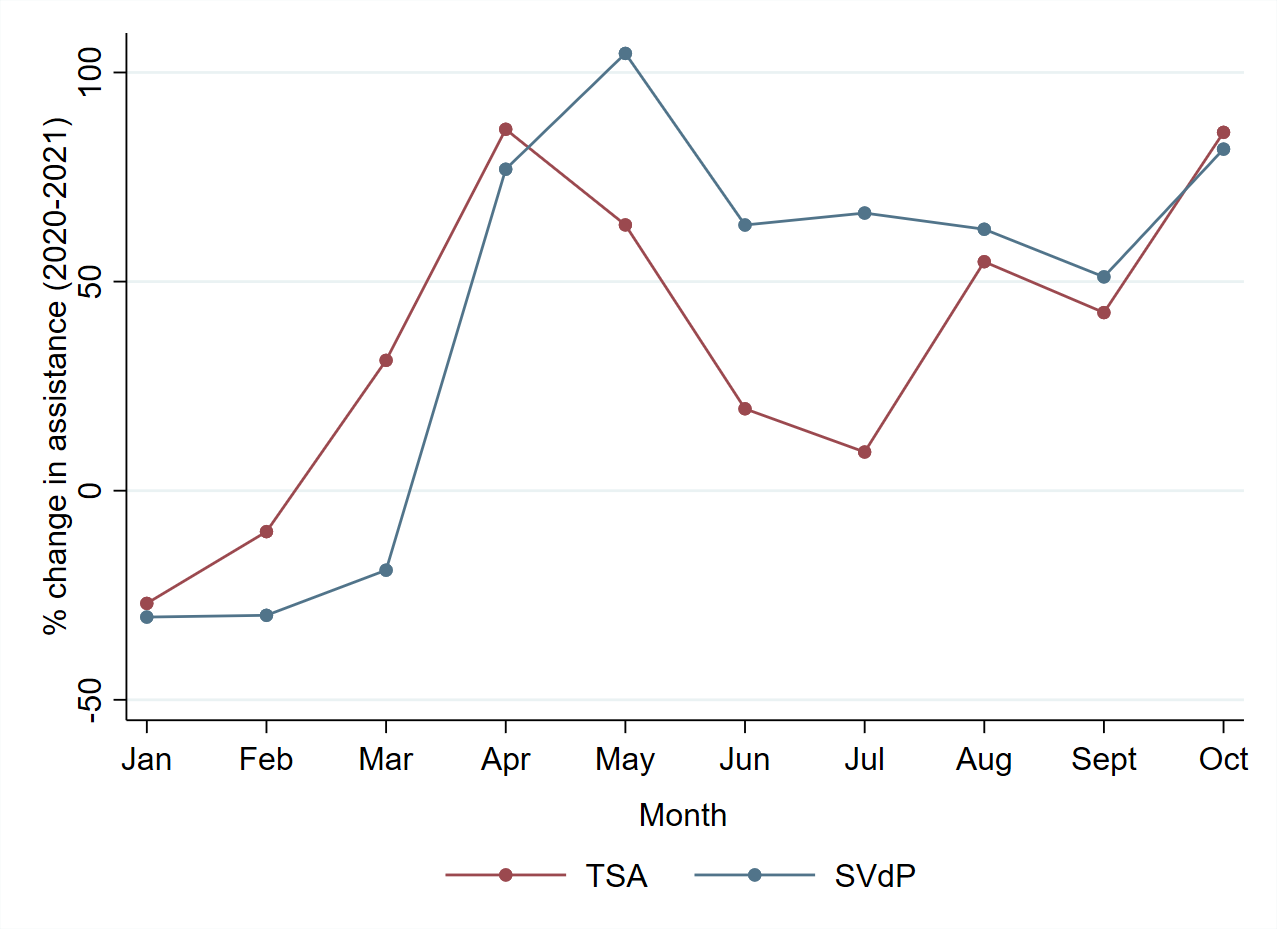


TSA – The Salvation Army; SVdP – St. Vincent de Paul Society Queensland.

**S6 Fig**. Non-parametric regression discontinuity plot

of the first AUD$750 Economic Support Payment

Time 0 pertains to 31 March 2020, the first day of the AUD$750 Economic Support Payment. The corresponding results of both parametric and non-parametric regression-discontinuity analyses are presented in Table S4.

**S7 Fig**. Trends in assistance records (2020 vs. previous years)


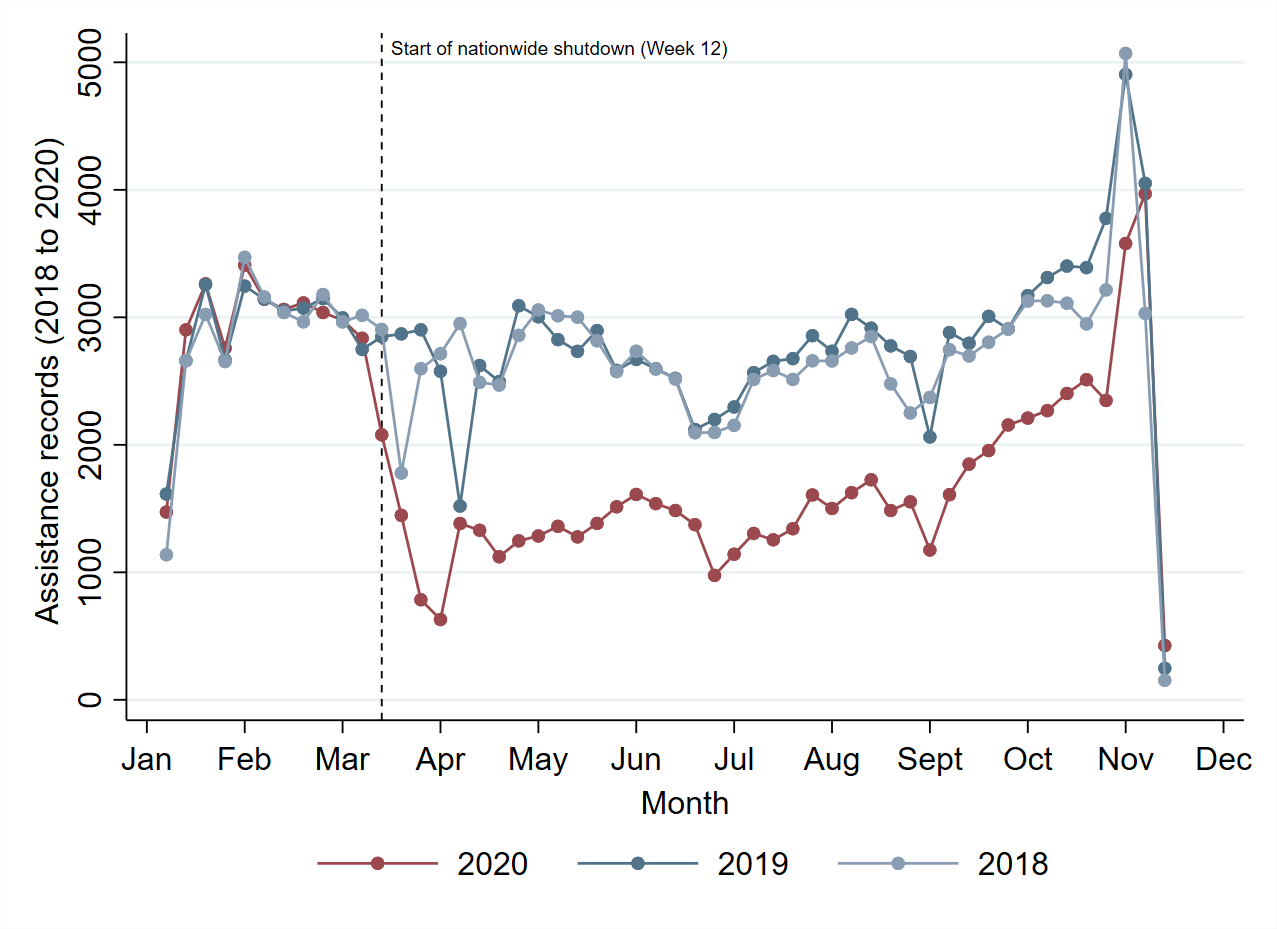


This figure combines the number of weekly assistance records from both charities.

**S8 Fig**. Coefficient plot from event study regression of time to and from

nationwide shutdown in March 2020


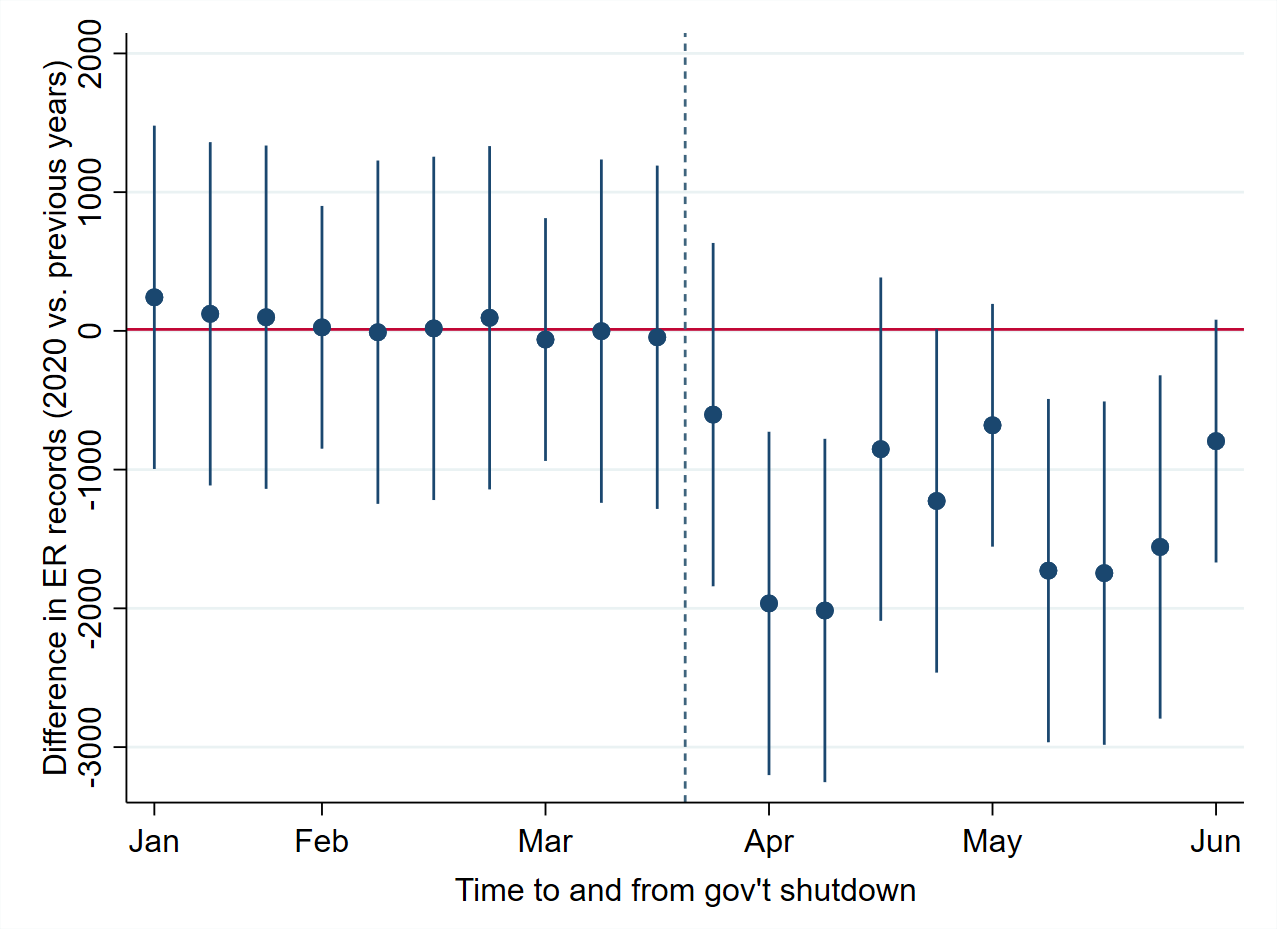


The dashed line denotes time 0, or the week of the nationwide shutdown. Whiskers denote 95%

confidence intervals.

**S1 Table**. Eligibility criteria for the Economic Support Payments and Coronavirus Supplement

| Payment type | Eligibility criteria |
| --- | --- |
| Economic Support Payment | The first AUD$750 Economic Support Payment was paid to individuals who were receiving eligible government income-support payments or who held an eligible concession card between 12 March and 13 April 2020. The following payments were included:  **Social-security income-support payments**   - Age Pension - Disability Support Pension - Carer Payment - Parenting Payment - Wife Pension - Widow B Pension - Austudy - Bereavement Allowance - Newstart Allowance - JobSeeker Payment - Youth Allowance - Partner Allowance - Sickness Allowance - Special Benefit - Widow Allowance   **Family-assistance payments**   - Family Tax Benefit, including the Double Orphan pension   **Veteran’s payments**   - Veteran Service Pension - Veteran Income Support Supplement - Veteran Compensation payments including lumpsum payments - War Widow(er) Pension - Veteran Payment   **Concession and health cards**   - Pensioner Concession Card - Commonwealth Seniors Health Card holders - Veteran Gold Card holders   **Other payments**   - ABSTUDY (Living Allowance) - Farm Household Allowance   The subsequent AUD$750 and AUD$250 Economic Support Payments were made available to the same recipients eligible for the first AUD$750 Economic Support Payment *with the exception of those who were receiving the Coronavirus Supplement*. |
| Coronavirus Supplement | Recipients of the following income-support payments were eligible for the AUD$550 Coronavirus Supplement:   - JobSeeker payment (including payments transitioning to JobSeeker such as Sickness Allowance, Wife Pension, and Bereavement Allowance) - Youth Allowance - Parenting Payment Partnered - Parenting Payment Single - Austudy - ABSTUDY Living Allowance - Farm Household Allowance - Special Benefit   Eligibility for the AUD$250 and AUD$150 Coronavirus Supplement remained unchanged. |

On 20 March 2020, JobSeeker replaced Newstart Allowance, Sickness Allowance, Bereavement Allowance a number of other payments.

Source: Adapted from Klapdor (2020).

**S2 Table.** Unstandardized coefficients from difference-in-difference

regressions of daily assistance records (Full results)

|  | β (Pooled) | β (SVdP) | β (TSA) |
| --- | --- | --- | --- |
| 2020×CS1 | –177.75^***^ | –139.51^***^ | –47.47^***^ |
|  | (16.37) | (14.87) | (7.49) |
| 2020×CS2 | –111.61^***^ | –90.65^***^ | –29.10^***^ |
|  | (19.67) | (17.61) | (8.05) |
| 2020×CS3 | –31.49^*^ | –37.53^**^ | 0.30 |
|  | (14.42) | (12.46) | (6.40) |
| 2020×ESP1 | –104.42^*^ | –96.81^*^ | –24.39† |
|  | (50.40) | (42.92) | (13.66) |
| 2020×ESP2 | 16.53 | 5.95 | 12.05 |
|  | (29.90) | (25.78) | (12.22) |
| 2020×ESP3 | –29.69 | –32.96 | 15.42 |
|  | (37.44) | (37.13) | (12.65) |
| 2021×ESP4 | –18.02 | –30.61^+^ | 19.47^**^ |
|  | (18.93) | (16.62) | (7.30) |
| 2020 | 5.82 | 2.39 | 0.07 |
|  | (14.72) | (13.71) | (5.96) |
| 2021 | –52.57^***^ | –43.95^***^ | –9.97^**^ |
|  | (7.78) | (7.35) | (3.75) |
| CS1 | 257.55^***^ | 180.92^***^ | 69.42^***^ |
|  | (32.52) | (33.65) | (9.76) |
| CS2 | –240.26^***^ | –172.60^***^ | –44.65^***^ |
|  | (32.45) | (33.68) | (11.52) |
| CS3 | 173.54^***^ | 129.55^***^ | –60.18^***^ |
|  | (14.67) | (12.47) | (11.55) |
| ESP1 | 177.89^***^ | 149.24^***^ | 18.04† |
|  | (37.72) | (38.82) | (9.62) |
| ESP2 | –117.77^***^ | –79.01^***^ | –37.18^***^ |
|  | (8.23) | (7.07) | (4.01) |
| ESP3 | 906.19^***^ | 777.32^***^ | 105.40^***^ |
|  | (12.98) | (12.69) | (8.78) |
| ESP4 | 97.98^**^ | 72.05^+^ | 123.85^***^ |
|  | (37.55) | (37.95) | (6.21) |
| Shutdown | 50.99^***^ | 35.94^***^ | 33.52^***^ |
|  | (8.57) | (6.95) | (4.29) |
| Lockdown | –4.33 | –12.69^*^ | 4.69^+^ |
|  | (6.60) | (6.30) | (2.84) |
| Holiday | –102.21^**^ | –25.38 | –86.80^***^ |
|  | (31.64) | (33.13) | (25.68) |
| Day-of-the-week fixed effects | Yes | Yes | Yes |
| Day-of-the-year fixed effects | Yes | Yes | Yes |
| Year fixed effects | Yes | Yes | Yes |
| N | 1,384 | 1,384 | 949 |
| R^2^ | 0.90 | 0.87 | 0.77 |

Clustered standard errors in parentheses. Statistical significance (two-sided tests): †*p*<0.10, ^*^*p*<0.05, ^**^*p*<0.01, ^***^*p*<0.001. SVdP – St. Vincent de Paul Society Queensland; TSA – The Salvation Army; CS1 – AUD$550 Coronavirus Supplement; CS2 – AUD$250 Coronavirus Supplement; CS3 – AUD$150 Coronavirus Supplement; ESP1 – 1^st^ AUD$750 Economic Support Payment; ESP2 – 2^nd^ AUD$750 Economic Support Payment; ESP3 – 3^rd^ AUD$250 Economic Support Payment; ESP4 – 4^th^ AUD$250 Economic Support Payment.

**S3 Table**. Unstandardized coefficients from Poisson difference-in-difference regressions

of daily assistance records (in Incidence Rate Ratios, IRR)

|  | β (Pooled) | β (SVdP) | β (TSA) |
| --- | --- | --- | --- |
| 2020×CS1 | 0.55^***^ | 0.51^***^ | 0.65^***^ |
|  | (0.02) | (0.02) | (0.03) |
| 2020×CS2 | 0.74^***^ | 0.72^***^ | 0.79^***^ |
|  | (0.04) | (0.05) | (0.04) |
| 2020×CS3 | 0.92^**^ | 0.89^**^ | 1.00 |
|  | (0.03) | (0.04) | (0.04) |
| 2020×ESP1 | 0.50^***^ | 0.43^***^ | 0.72^**^ |
|  | (0.06) | (0.07) | (0.08) |
| 2020×ESP2 | 0.94 | 0.85^**^ | 1.08 |
|  | (0.07) | (0.05) | (0.11) |
| 2020×ESP3 | 0.96 | 0.90 | 1.15 |
|  | (0.18) | (0.22) | (0.10) |
| 2021×ESP4 | 0.93† | 0.88^**^ | 1.15^**^ |
|  | (0.03) | (0.04) | (0.06) |
| 2020 | 0.99 | 0.99 | 1.01 |
|  | (0.03) | (0.03) | (0.04) |
| 2021 | 0.87^***^ | 0.85^***^ | 0.93^**^ |
|  | (0.02) | (0.02) | (0.02) |
| CS1 | 2.27^***^ | 2.18^***^ | 1.70^***^ |
|  | (0.25) | (0.27) | (0.12) |
| CS2 | 0.07^***^ | 0.10^***^ | 0.03^***^ |
|  | (0.01) | (0.01) | (0.00) |
| CS3 | 0.03^***^ | 0.05^***^ | 0.17^***^ |
|  | (0.00) | (0.00) | (0.01) |
| ESP1 | 2.31^***^ | 2.50^***^ | 1.24^**^ |
|  | (0.27) | (0.33) | (0.09) |
| ESP2 | 0.74^***^ | 0.75^***^ | 0.72^***^ |
|  | (0.02) | (0.02) | (0.02) |
| ESP3 | 90.57^***^ | 91.56^***^ | 54.44^***^ |
|  | (9.73) | (13.61) | (3.53) |
| ESP4 | 67.86^***^ | 46.73^***^ | 9.90^***^ |
|  | (8.39) | (6.80) | (0.41) |
| Shutdown | 1.03^**^ | 0.96^**^ | 1.19^***^ |
|  | (0.01) | (0.01) | (0.02) |
| Lockdown | 0.87^***^ | 0.84^***^ | 1.03 |
|  | (0.02) | (0.02) | (0.02) |
| Holiday | 0.67^***^ | 0.90 | 0.34^*^ |
|  | (0.07) | (0.11) | (0.15) |
| Day-of-the-week fixed effects | Yes | Yes | Yes |
| Day-of-the-year fixed effects | Yes | Yes | Yes |
| Year fixed effects | Yes | Yes | Yes |
|  |  |  |  |
| N | 1,384 | 1,384 | 949 |

Clustered standard errors in parentheses. Statistical significance (two-sided tests): †*p*<0.10, ^*^*p*<0.05, ^**^*p*<0.01, ^***^*p*<0.001. SVdP – St. Vincent de Paul Society Queensland; TSA – The Salvation Army; CS1 – AUD$550 Coronavirus Supplement; CS2 – AUD$250 Coronavirus Supplement; CS3 – AUD$150 Coronavirus Supplement; ESP1 – 1^st^ AUD$750 Economic Support Payment; ESP2 – 2^nd^ AUD$750 Economic Support Payment; ESP3 – 3^rd^ AUD$250 Economic Support Payment; ESP4 – 4^th^ AUD$250 Economic Support Payment.

**S4 Table.** Unstandardized coefficients from

panel fixed effects regressions of daily assistance records

|  | β (Pooled) | β (SVdP) | β (TSA) |
| --- | --- | --- | --- |
| CS1 | -177.70*** | -139.47*** | -45.71*** |
|  | (14.02) | (12.73) | (6.13) |
| CS2 | -111.54*** | -90.60*** | -25.35*** |
|  | (16.83) | (15.07) | (6.80) |
| CS3 | -29.17* | -35.80*** | 2.44 |
|  | (12.42) | (10.68) | (5.09) |
| ESP1 | -104.61* | -96.96** | -21.86* |
|  | (43.04) | (36.66) | (10.60) |
| ESP2 | 16.60 | 6.00 | 12.66 |
|  | (25.53) | (22.01) | (9.57) |
| ESP3 | -29.65 | -32.93 | 14.93 |
|  | (32.02) | (31.75) | (11.06) |
| ESP4 | -20.40 | -32.39* | 14.52* |
|  | (16.29) | (14.27) | (6.16) |
|  |  |  |  |
| Shutdown controls | Yes | Yes | Yes |
| Lockdown controls | Yes | Yes | Yes |
| Holiday controls | Yes | Yes | Yes |
| Day-of-the-week effects | Yes | Yes | Yes |
| Year effects | Yes | Yes | Yes |
|  |  |  |  |
| N | 1,384 | 1,384 | 942 |
| R^2^ | 0.85 | 0.80 | 0.60 |

Clustered standard errors in parentheses. Statistical significance (two-sided tests): †*p*<0.10, ^*^*p*<0.05, ^**^*p*<0.01, ^***^*p*<0.001. SVdP – St. Vincent de Paul Society Queensland; TSA – The Salvation Army; CS1 – AUD$550 Coronavirus Supplement; CS2 – AUD$250 Coronavirus Supplement; CS3 – AUD$150 Coronavirus Supplement; ESP1 – 1^st^ AUD$750 Economic Support Payment; ESP2 – 2^nd^ AUD$750 Economic Support Payment; ESP3 – 3^rd^ AUD$250 Economic Support Payment; ESP4 – 4^th^ AUD$250 Economic Support Payment.

**S5 Table**. Regression-discontinuity estimates for the

first AUD$750 Economic Support Payment

|  | Parametric | | Non-parametric | |
| --- | --- | --- | --- | --- |
| Discontinuity (31 Mar 2020) | –13.86^*^ | –12.93^*^ | –13.09^***^ | –16.55^***^ |
|  | (5.99) | (5.57) | (3.57) | (2.81) |
| Constant | 23.93^***^ | 37.64^***^ | - | - |
|  | (4.18) | (8.03) |  |  |
|  |  |  |  |  |
| Controls | No | Yes | No | Yes |
| N | 36 | 36 | 46 | 31 |
| R^2^ | 0.57 | 0.65 | - | - |

Standard errors in parentheses. Statistical significance (two-sided tests): †*p*<0.10, ^*^*p*<0.05, ^**^*p*<0.01, ^***^*p*<0.001.

Parametric models are based on a polynomial of order one (i.e., a linear model) with a bandwidth ranging from −29 (02 March 2020) to 20 (20 Apr 2020). The bandwidth excludes the beginning of the Coronavirus Supplement. Higher-order polynomials of orders two and three were not statistically significant and hence not included in the final model. For the non-parametric model, N pertains to the effective number of observations. Controls include holidays, shutdown dates, and day-of-the-week effects.

**S6 Table**. Unstandardized coefficients from triple-difference regression of charitable assistance

using The Salvation Army data (Full results)

|  | β |
| --- | --- |
| 2020×CS1×Eligible | –21.93^***^ |
|  | (3.78) |
| 2020×CS2×Eligible | –19.14^***^ |
|  | (4.71) |
| 2021×CS3×Eligible | –0.99 |
|  | (3.43) |
| 2020×ESP1×Eligible | –3.02 |
|  | (5.45) |
| 2020×ESP2×Eligible | –1.73 |
|  | (4.08) |
| 2020×ESP3×Eligible | –2.36 |
|  | (9.57) |
| 2021×ESP4×Eligible | 3.47 |
|  | (4.02) |
| 2020×CS1 | –0.51 |
|  | (1.50) |
| 2020×CS2 | –1.12 |
|  | (1.76) |
| 2021×CS3 | –0.25 |
|  | (1.72) |
| 2020×ESP1 | 2.38 |
|  | (3.65) |
| 2020×ESP2 | 0.63 |
|  | (2.45) |
| 2020×ESP3 | 0.27 |
|  | (3.32) |
| 2021×ESP4 | 1.01 |
|  | (2.89) |
| CS1×Eligible | 2.03 |
|  | (3.46) |
| CS2×Eligible | 5.23 |
|  | (4.13) |
| CS3×Eligible | –0.97 |
|  | (3.41) |
| ESP1×Eligible | –6.80^+^ |
|  | (4.01) |
| ESP2×Eligible | 0.84 |
|  | (3.00) |
| ESP3×Eligible | 5.15 |
|  | (6.80) |
| ESP4×Eligible | –4.11 |
|  | (2.90) |
| Eligible | 25.25^***^ |
|  | (3.38) |
| 2020 | 0.05 |
|  | (1.25) |
| 2021 | –0.54 |
|  | (0.94) |
| CS1 | 5.73^***^ |
|  | (1.64) |
| CS2 | –3.58^+^ |
|  | (1.94) |
| CS3 | –1.66 |
|  | (1.66) |
| ESP1 | 6.41^**^ |
|  | (2.11) |
| ESP2 | 0.48 |
|  | (1.57) |
| ESP3 | 12.86^***^ |
|  | (2.93) |
| ESP4 | 10.44^***^ |
|  | (1.39) |
|  |  |
| Shutdown/lockdown/holiday controls | Yes |
| Day-of-the-week | Yes |
| Day-of-the-year effects | Yes |
| Year fixed effects | Yes |
|  |  |
| N | 2,682 |
| R^2^ | 0.66 |

Clustered standard errors in parentheses. Statistical significance (two-sided tests): †*p*<0.10, ^*^*p*<0.05, ^**^*p*<0.01, ^***^*p*<0.001. CS1 – AUD$550 Coronavirus Supplement; CS2 – AUD$250 Coronavirus Supplement; CS3 – AUD$150 Coronavirus Supplement; ESP1 – 1^st^ AUD$750 Economic Support Payment; ESP2 – 2^nd^ AUD$750 Economic Support Payment; ESP3 – 3^rd^ AUD$250 Economic Support Payment; ESP4 – 4^th^ AUD$250 Economic Support Payment. *Eligible* refers to records from individuals who were eligible for the Coronavirus Supplement and Economic Support Payments.

**S7 Table.** Day and month corresponding to the payment periods

| Payment | Dates |
| --- | --- |
| CS1 | 27 April – 24 September |
| CS2 | 25 September – 31 December |
| CS3 | 1 January – 31 March |
| ESP1 | 31 March – 14 April |
| ESP2 | 13 July – 27 July |
| ESP3 | 30 November – 14 December |
| ESP4 | 1 March – 15 March |

CS1 – AUD$550 Coronavirus Supplement; CS2 – AUD$250 Coronavirus Supplement; CS3 – AUD$150 Coronavirus Supplement; ESP1 – 1^st^ AUD$750 Economic Support Payment; ESP2 – 2^nd^ AUD$750 Economic Support Payment; ESP3 – 3^rd^ AUD$250 Economic Support Payment; ESP4 – 4^th^ AUD$250 Economic Support Payment.
