# Supplementary material for: Increases in income-support payments reduce the demand for charity: A difference-in-difference analysis of charitable-assistance data from Australia over the COVID-19 pandemic
Source: PLoS One. 2023 Jul 12;18(7):e0287533. doi: 10.1371/journal.pone.0287533 (PMC10337872; doi:10.1371/journal.pone.0287533)
Supplement: S2 File — (PDF) [file pone.0287533.s002.pdf]

Requests for Emergency Relief from St. Vincent de Paul Society Queensland and The Salvation Army, 2018-2021

Data notes

Overview of the data

This dataset contains the number of requests for emergency relief from the two largest emergency relief providers in the state of Queensland, Australia - the St. Vincent de Paul Society Queensland the The Salvation Army. The data covers the time period between 2018 to 2021. The dataset has been used to analyse the impact of increased government income support during COVID-19 on the demand for emergency relief. Findings from the analysis show that the increased support provided by government in 2020 led to reduced demand for emergency relief.

This dataset has been created based on administrative data held by St. Vincent de Paul Society Queensland and The Salvation Army. In particular, all requests for emergency relief in the state of Queensland, Australia between 2018 to 2021 have been aggregated at the date level to create a time series dataset.

Overview of the datasets

Two files have been deposited through this repository, as follows:

- Combined_ER_2018_2021 – This dataset contains the combined number of emergency relief requests for both organisations from 2018 to 2021. This was used for descriptive analysis as well as a Difference-in-Difference analysis.
- Salvos_ER_byincgroup – This dataset only contains requests for emergency relief from The Salvation Army. The number of requests per day have been broken down into various income groupings. This particular dataset was used for triple difference analysis.

Data dictionary

Combined_ER_2018_2021

**Variable name**	**Description**
Visit date	Date of ER request
ER provider	The organisation providing emergency relief.<br>Vinnies – St. Vincent de Paul Society Queensland<br>Salvos – The Salvation Army
Number of ER requests	The number of emergency relief requests for a given date

Salvos_ER_byincgroup

**Variable name**	**Description**
Visit date	Date of ER request
5 income groups	The income source of people requesting emergency relief. There are five main income groupings:<br>- Receiving income support and eligible for Covid Supplement (JS/SA/YA/PP/PA/FHA)<br>- Receiving other income support and ineligible for Covid Supplement<br>- Not receiving income support (international students, temporary migrants, asylum seekers, other non-citizen/Permanent Resident)<br>- Not receiving income support but have other income (e.g. pension, employment)<br>- Unknown/Other/Not stated/No income
Daily # of visits	The number of emergency relief requests for a given date and income group

JS – JobSeeker; SA – Special Allowance; YA – Youth Allowance; PP – Parenting Payment; Partner Allowance - ; FHA – Farm Household Allowance
